# Rarely acquired type II-A CRISPR-Cas spacers mediate anti-viral immunity through the targeting of a non-canonical PAM sequence

**DOI:** 10.1093/nar/gkad501

**Published:** 2023-06-09

**Authors:** Claire T Kenney, Luciano A Marraffini

**Affiliations:** Laboratory of Bacteriology, The Rockefeller University, 1230 York Ave, New York, NY 10065, USA; Laboratory of Bacteriology, The Rockefeller University, 1230 York Ave, New York, NY 10065, USA; Howard Hughes Medical Institute, The Rockefeller University, 1230 York Ave, New York, NY 10065, USA

## Abstract

The *Streptococcus pyogenes* type II-A CRISPR-Cas systems provides adaptive immunity through the acquisition of short DNA sequences from invading viral genomes, called spacers. Spacers are transcribed into short RNA guides that match regions of the viral genome followed by a conserved NGG DNA motif, known as the PAM. These RNA guides, in turn, are used by the Cas9 nuclease to find and destroy complementary DNA targets within the viral genome. While most of the spacers present in bacterial populations that survive phage infection target protospacers flanked by NGG sequences, there is a small fraction that target non-canonical PAMs. Whether these spacers originate through accidental acquisition of phage sequences and/or provide efficient defense is unknown. Here we found that many of them match phage target regions flanked by an NAGG PAM. Despite being scarcely present in bacterial populations, NAGG spacers provide substantial immunity *in vivo* and generate RNA guides that support robust DNA cleavage by Cas9 *in vitro*; with both activities comparable to spacers that target sequences followed by the canonical AGG PAM. In contrast, acquisition experiments showed that NAGG spacers are acquired at very low frequencies. We therefore conclude that discrimination against these sequences occurs during immunization of the host. Our results reveal unexpected differences in PAM recognition during the spacer acquisition and targeting stages of the type II-A CRISPR-Cas immune response.

## Graphical Abstract

**Graphical Abstract:**
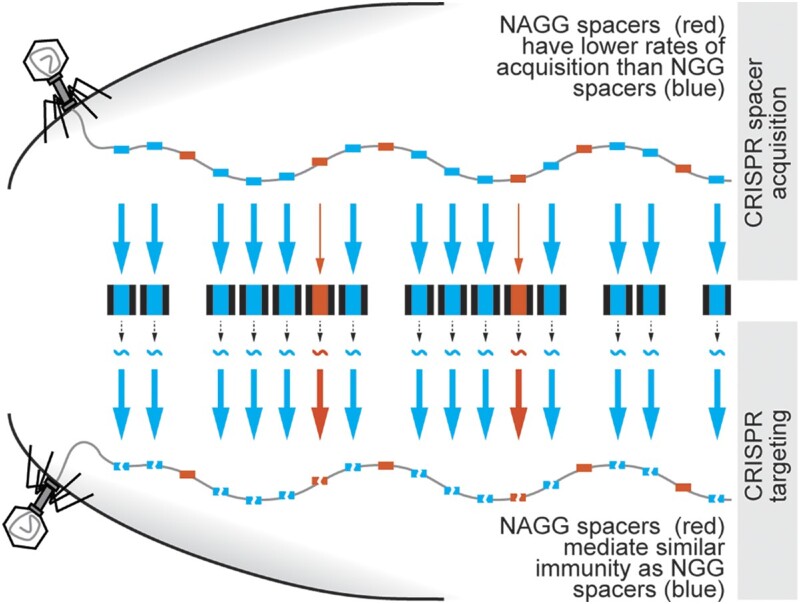

## INTRODUCTION

Clustered, regularly interspaced, short, palindromic repeats (CRISPR) loci and their associated (*cas*) genes protect bacteria and archaea from viral (phage) predation through a mechanism that confers adaptive immunity to these organisms (1). The CRISPR-Cas response to phage infection is divided into two phases. In the immunization phase, Cas integrases extract a short fragment of the invading phage DNA, known as a ‘spacer’, and insert it between the repeats of the CRISPR locus (2). This allows the bacterial host to collect a memory of the infection that will be used during the second phase of the CRISPR response, known as the targeting phase, to recognize the phage in a subsequent exposure. This is achieved first through the transcription and processing of the spacer into a short RNA guide (3–5), known as the CRISPR RNA (crRNA), which forms a ribonucleoprotein complex with crRNA-guided Cas nucleases (6–9). These nucleases use the crRNA to find complementary nucleic acids (known as protospacers) of the invading phage and cleave them, thus limiting phage replication and propagation (10–12).

Depending on the *cas* gene content, CRISPR-Cas systems are classified into six different types (13) that diverge in the general molecular mechanism of immunity described above. Type II CRISPR-Cas systems are defined by the presence of the signature gene *cas9*, which encodes a crRNA-guided nuclease that plays key roles in both the immunization and targeting phases of immunity. Spacers incorporated into the type II CRISPR locus match phage regions that are flanked by a conserved sequence, known as the protospacer-adjacent motif (PAM) (14,15). For one of the most studied of these systems, the type II-A system of *Streptococcus pyogenes* SF370, the PAM sequence is NGG (4,16). Spacer acquisition in this system requires Cas9, the Cas1/Cas2 integrase and the DNA-sliding hexameric complex formed by Csn2 (17,18). These form a ‘supercomplex’ that selects and integrates DNA sequences from invading phages (19), preferably from free DNA ends (19–22). Cas9 plays two different roles in this process. First, it uses its PAM binding-recognition domain to select phage DNA fragments that contain NGG motifs (17). This recognition is achieved by two arginine residues, each of which establishes sequence-specific hydrogen-bonding interactions with one of the guanosines of the GG PAM dinucleotide (23). Second, Cas9 performs the nucleolytic cleavage of these phage DNA fragments to 30 nucleotides, the length of the spacers in the type II-A CRISPR array (19).

During targeting, *S. pyogenes* Cas9 uses the crRNA-guide to locate a complementary sequence in the phage DNA and cleave it (7). Target recognition is first achieved by searching for GG dinucleotides on the phage DNA (24). Cas9 interaction with a potential PAM results in the partial melting of the DNA upstream of the motif, which allows an attempt at annealing the crRNA to the bottom strand of the target DNA (24). First a region of 6–8 nucleotides known as the seed sequence is paired. If this RNA:DNA hybrid is formed, then the rest of the crRNA sequence is paired to the single stranded DNA (ssDNA) target (24). When a full R-loop structure is formed due to pairing, the two nuclease domains of Cas9, HNH and RuvC, each cleave a DNA strand of the target, generating a double strand break in the viral DNA (7). While this break is genotoxic for the phage and prevents its replication and propagation (10), the mechanism has weaknesses that can be exploited to circumvent type II-A CRISPR-Cas immunity. Mutant phages present in the population (usually at a very low frequency and known as ‘escapers’) contain nucleotide modifications in either the PAM or seed sequence, and can thereby escape Cas9 cleavage and kill the host cell (15,22). In addition to its role during the targeting phase of type II-A immunity, Cas9’s nuclease activity has been repurposed to cleave genomic DNA of eukaryotic cells for the development of revolutionary, sequence-specific gene editing techniques (25,26).

Due to Cas9’s ability to recognize NGG PAM sequences during both the immunization and targeting phases, the vast majority of spacers present in bacterial populations that survive phage infection target protospacers flanked by these sequences, and only a very small fraction (0.03%) target non-canonical PAMs (17). Whether these spacers originate by accidental acquisition of phage sequences and/or provide efficient defense is unknown. Here, we investigated these rare spacers and found that many of them match phage target regions flanked by an NAGG PAM. We determined that, despite being scarcely present in bacterial populations, NAGG spacers provide substantial immunity *in vivo* and generate crRNA guides that support robust DNA cleavage by Cas9 *in vitro*; with both activities comparable to spacers that target sequences followed by the canonical AGG PAM. This is also the case for NAGG spacers that are not found in the spacer repertoire of surviving bacterial populations. In contrast, acquisition experiments showed that NAGG spacers are acquired at very low frequencies. We therefore conclude that discrimination against these sequences occurs during immunization of the host. Our results reveal unexpected differences in PAM recognition during the two stages of the type II-A CRISPR-Cas immune response.

## MATERIALS AND METHODS

### Plasmid construction

The plasmids used in this study are listed in Supplementary File 2, as are the sequences of oligonucleotides used in this study. For all experiments, some or all of the *S. pyogenes* type II-A CRISPR-Cas locus was cloned into staphylococcal vector plasmids, which were expressed within *Staphylococcus aureus* RN4220 cells. The assays investigating targeting used *S*.*aureus* RN220 cells that carried plasmids derived from the parent plasmid, pDB114, which was modified to contain a repeat-spacer-repeat CRISPR array with a defined spacer. To create these plasmids, we used a restriction digest-based cloning approach previously described (27).

In short, the parent plasmid pDB441, contains a chloramphenicol-resistance cassette, *tracr*, *cas9*, leader sequence, and two CRISPR repeats flanking a 30 bp sequence housing two *bsaI* restriction sites. pDB114 was mixed with the BsaI-HFv2 restriction enzyme (NEB) and incubated at 37°C for 4–6 h. Two IDT single stranded DNA (ssDNA)oligonucleotides (oligos) (a ‘‘forward” DNA oligo and a “reverse” DNA oligo) were phosphorylated with PNK (NEB) at 37°C for 30-60 min. After phosphorylation, the oligos were annealed by adding NaCl and incubating for 5 min at 98°C, then allowing the reaction to cool to room temperature (∼2.5 h). The annealed double-stranded DNA (dsDNA) oligos were diluted 1:10 in nuclease-free water and ligated to the digested plasmid in a 20 μl reaction at room temperature overnight.

Reactions were drop dialyzed for 30 min in ddH_2_O and transformed into electrocompetent *S. aureus* RN4220 cells. 500 μl BHI was added to the transformed cells, which were then incubated at 37°C for 1–2 h with agitation, spread onto agar plates of BHI and 10 μg/ml chloramphenicol, and incubated overnight at 37°C. Resulting colonies were confirmed for correct plasmid construction by dissolving in 20 μl colony lysis buffer (25 mM HEPES, 150 mM KCl, 5% (v/v) glycerol, 20% sucrose) (28), and 1 μl lysostaphin (100 μg/mL final, Ambi Products LLC), incubating for 20 min at 37°C and then 10 min at 98°C, amplifying the CRISPR array via PCR using one of the IDT ssDNA oligos, and sending the samples for Sanger sequencing.

### Bacterial strains and growth conditions

Growth of of *S. aureus* RN4220 (29) cultures was carried out in Bacto brain-heart infusion (BHI) broth medium at 37°C with agitation at 220 RPM. Liquid experiments were carried out in 3 ml BHI medium in 15-ml conical tubes unless otherwise noted. Wherever applicable, media was supplemented with 10 μg/ml chloramphenicol to ensure maintenance of plasmids derived from the chloramphenicol-resistant staphylococcal vector plasmid, pC194 (30). This includes pDB114, its derivative plasmids, pGG32-ΔtrL, and pWJ40 (described below). For RN4220::pKL55-iTET-B1 strains, which contain the kanamycin resistance gene integrated into the RN4220 chromosome (31), BHI was also supplemented with 25 μg/ml kanamycin for chromosomal marker selection. See Supplementary File 2 for a full list of bacterial strains, plasmids, and oligonucleotides used in this study.

### Bacteriophage propagation

The previously constructed, lytic *S. aureus* phage, ФNM4γ4 (17), and mutant phages derived from ФNM4γ4, were used throughout the study. Phage strains were amplified by first launching overnight cultures of *S. aureus* RN4220 cells. The following day, the culture was diluted 1:100 in fresh BHI, supplemented with 5 mM CaCl_2_ and the appropriate antibiotic, and grown at 37°C with agitation to an optical cell density (OD_600_) of 0.2–0.6 (about 1 h and 10 min). The culture was rediluted to a normalized OD_600_ of 0.2, phage was added at a multiplicity of infection (MOI) of 0.1, and the cultures were grown for an additional 4 h with agitation at 37°C. Then, cultures were spun down for 5 min at 4300 RPM. The lysates were filtered through 0.45 μm syringe filters (Acrodisc) and stored in BHI at 4°C. Plaque formation assays were conducted to assess the number of infectious phage particles in the resulting stocks. In subsequent phage infection assays, an MOI = 1 was used unless otherwise noted, and BHI media was supplemented with the appropriate antibiotic and 5 mM CaCl_2_ to facilitate phage adsorption.

### Phage mutant construction

We employed phage recombination techniques using plasmids to create ФNM4γ4 phages with point mutations in different nucleotides of the NAGG PAMs (the A2, G3 or G4 of the N1-A2-G3-G4 sequence) corresponding to *spc11, spc14, spc15* and *spc17* (22). See Supplementary File 2 for a full list of plasmids and oligonucleotides used to generate phage mutants in this study.

The phages with a mutation in G3 or G4 were isolated as spontaneous escaper plaques following infection of WT ФNM4γ4 on a soft agar lawn of *S. aureus* RN4220 cells carrying plasmids with the NAGG or AGG version of the spacer of interest. Single plaques were isolated and re-passaged to single plaques on lawns of the same strain in a plaque formation assay (see protocol below). Phage DNA was extracted by boiling the phage (98°C, 10 min), PCR amplified, and Sanger sequenced to identify the plaques with phage harboring a mutation of interest.

No escapers isolated from NAGG *spc* strains or AGG *spc* strains had A2 mutations. To obtain such phage mutants, plasmids were constructed, containing a ∼1 kb homology with the phage that included an A to C mutation in the NAGG of interest, and a G to C mutation in the G3 of an upstream, non-essential NGG PAM. WT ФNM4ɣ4 was passaged on the resulting strain on soft agar. To isolate recombinant phages, plaques were picked and re-passaged on soft agar to single plaques. Phages from those plaques were then passaged on strains containing plasmids with a spacer targeting only the upstream protospacer with the mutated NCG PAM. The phages isolated from the resulting plaques were amplified and sequenced to confirm the A to C mutation in the NAGG of interest.

### Preparation of electrocompetent *S. aureus* RN4220 strains

Electrocompetent *S. aureus* RN4220 cells were prepared and transformed with DNA plasmids using a previously described method (31), with the exception of using BHI medium instead of TSB medium.

### Plasmid miniprep

Unless otherwise indicated, 1–3 ml of an overnight *S. aureus* RN4220 culture, were pelleted and resuspended in 250 μl Buffer P1. 10 μl Lysostaphin (Ambi Products LLC, 100 μg/ml final) was added, and the cells were incubated with agitation at 37°C for 30–60 min. Following lysis, plasmids were isolated using the QIAGEN Spin Miniprep kit according to the manufacturer's protocol, beginning with addition of P2. DNA was eluted from each column in 40 μl Millipore water.

### Enumeration of colony forming units (CFU)

To determine the concentration of bacteria that survived phage infection in liquid culture, ten-fold dilutions of *S. aureus* RN4220 strains were spotted on 50% BHI agar plates supplemented with the appropriate antibiotic. The plates were incubated overnight at 37°C and colony-forming units (CFU) were enumerated the next day (20).

### Enumeration of plaque forming units (PFU)

Phage titer assays were performed as previously described (31). In brief, serial dilutions of the ФNM4γ4 or mutant ФNM4γ4 stock were prepared in triplicate and spotted on fresh 50% BHI top agar lawns containing the appropriate antibiotic, 5 mM CaCl_2_ and RN4220 cells harboring a type II-A CRISPR plasmid targeting the phage. Plates were incubated at 37°C overnight, and resulting plaques were enumerated the next day to calculate plaque forming units (PFU).

### Growth curves of bacterial infection

Bacterial infection growth was measured as previously described (31) with minor alterations. Overnight cultures were launched and the following day, they were diluted 1:100 in fresh BHI, supplemented with 5 mM CaCl_2_ and the appropriate antibiotic, and grown at 37°C with agitation to OD_600_ = 0.2–0.6 (about 1 h and 10 min). The culture was normalized to OD_600_ = 0.2, in triplicate. Phage was added at an MOI = 1 (unless noted otherwise), and the cultures were loaded into a 96-well plate (Cellstar). The plate was incubated at 37°C with agitation for 18–24 h, with OD measurements taken every ten min (by a Tecan Infinite 200 PRO) to generate growth curves.

### *In vitro* cas9 target cleavage assay

*In vitro* cleavage by Cas9 was tested following a previously published protocol (22) with minor alterations. A 1 kb double stranded DNA (dsDNA) target, containing the spacer and PAM of interest, was generated by mixing 20 μl colony lysis buffer with 1 μl ФNM4γ4 stock and heating the mixture to 98°C for 10 min. Samples were spun for 1–2 min at 15000 RPM. Four PCRs were run for each sample, each with 1 μl of the supernatant of the spun phage DNA and 2.5 μl of each 10 μM forward and reverse oligos. The oligos used were oCK124 and oCK125 for *spc11*, oCK126 and oCK127 for *spc14*, oCK028 and oCK029 for *spc15*, and oCK140 and oCK131 for *spc17*. For each sample, the four PCRs were combined and run through one DNA Clean & Concentrator^TM^-5 column in the Zymo Research kit, according to the manufacturer's protocol. DNA was eluted in 6 μl nuclease-free H_2_O and diluted to 100 nM.

crRNAs were ordered from IDT to correspond with either the NAGG or AGG versions of *spc11, spc14, spc15* and *spc17* (RNA oligos listed in Supplementary File 2). Each was diluted to 100 μM in IDTE buffer. 1 μl of crRNA was mixed at a 1:1 ratio with 1 μl 100 μM tracrRNA (IDT), and 8 μl DNA duplex buffer (NEB) to form tracrRNA:crRNA RNA duplexes. The mixture was heated to 95°C for 5 min and cooled via thermocycler at −0.6°C per minute, until reaching room temperature (20°C, amounting to ∼2.5 h). The RNA duplexes were diluted 1:10 in nuclease-free water and used immediately in the cleavage reactions.

Cleavage assays were performed much as described previously (22) at Cas9:crRNA:tracrRNA concentrations of 0, 6.25, 12.5, 25, 50, 100 and 200 nM. Briefly, 20 μM Cas9 (NEB) was diluted to 1 μM by mixing 1.5 μl 20 μM Cas9, 3 μl 10× NEB Cas9 reaction buffer (NEB), and 25.5 μl nuclease-free H_2_O. Then the 1 μM Cas9 was mixed 1:1 with 1 μM tracrRNA:crRNA (50 μl nuclease-free H_2_O, 12.5 μl 10× NEB Cas9 reaction buffer, 25 μl 1 μM Cas9 and 25 μl 1 μM tracrRNA:crRNA) and incubated at room temperature for 10 min. Six serial, 2-fold dilutions were made by diluting the resulting ribonucleoprotein (RNP) mixture in nuclease free H_2_O. 5 μl of the 100 nM DNA substrate was incubated with 45 μl of the RNP dilutions for a total reaction volume of 50 μl and a final DNA concentration of 10 nM. All reactions were incubated at 37°C for 5 min before being quenched with 1 μl proteinase K (NEB). Samples were stored at –20°C.

For analysis, samples were brought to room temperature and diluted 10-fold in nuclease-free H_2_O. Cleavage products were visualized and quantified by automated gel electrophoresis and imaging using a Tapestation 4200 bioanalyzer (Agilent), and following the manufacturer's protocol for the Agilent D500 ScreenTape Assay (Agilent).

### NAGG and AGG spacer library generation

To generate the NAGG and AGG spacer libraries, a previously published protocol was used (32), with the following modifications. Two libraries of CRISPR plasmids derived from pDB114 were generated, one containing all 787 spacers derived from all NAGG PAMs that are found in ФNM4γ4 (NAGG spacer library), and the other containing all 787 spacers derived from all AGG PAMs in ФNM4γ4 (AGG spacer library). For each NAGG and AGG spacer library, 787 oligonucleotides (85 nucleotides in length), were purchased from Twist Biosciences. Each oligo contained a unique ФNM4γ4-matching spacer, repeat homology, *bsaI* sites, and universal priming sites. The library was made double-stranded by PCR and the product was purified by phenol-chloroform extraction. Individual spacers from each annealed oligo were introduced into pDB114 via Golden Gate cloning with BsaI-HFv2 (NEB) and T7 DNA ligase (NEB), then electroporated into RN4220 electrocompetent cells. To obtain at least 10× coverage for each spacer in the library, 8000 colonies were pooled, pelleted, resuspended in 1 ml 10% DMSO, aliquoted, and stored at -80°C. Complete coverage of the NAGG and AGG spacer libraries was confirmed via next generation DNA sequencing, as described below.

### Next generation DNA sequencing

Plasmid DNA was extracted from liquid cultures after phage infection experiments (see protocols above) and used as a template for Phusion PCR to amplify the CRISPR array. After bands of the CRISPR array were extracted and purified from a 2% agarose gel, samples were prepared for sequencing with the TrueSeq Nano DNA Library Prep protocol (Illumina) and subject to Illumina MiSeq sequencing. Data analysis was performed in Python, similar to that described previously (33). In short, the spacer sequences were extracted and the number of reads for each spacer was recorded from the raw Illumina FASTQ files. For each unique spacer sequence matching the ФNM4γ4 genome, various characteristics were determined and recorded, including the spacer's frequency within the sequencing population (number of reads of that spacer compared to total spacer reads sequenced), the position of its protospacer in the ФNM4γ4 genome, the strand of the genome on which the protospacer is found, and the PAM sequence flanking the protospacer (see Supplementary File 1).

### Spacer acquisition from electroporated dsDNA oligonucleotides

To study the acquisition of spacers from transformed DNA oligos, electrocompetent *S. aureus* RN4220 cells were made by the protocol above, except that the overnight culture was diluted 1:500 (400 μl overnight culture, 200 ml BHI, 200 μl cm10), resulting in about 2–2.5 h to regrow to OD_600_ = 0.4. To further increase the cells' competence, 0.5 M sucrose was used to perform three washes, instead of two washes in 10% glycerol (34). The RN4200 strain carried the plasmid, pGG32-ΔtrL, that contains a CRISPR array of a single repeat, and has the long *tracr* promoter sequence deleted. This deletion results in an increase rate of spacer acquisition (27), which is otherwise relatively inefficient from transformed dsDNA (35).

To create dsDNA oligos to transform into the pGG32-ΔtrL strain, 63 bp amplicons from ФNM4γ4 were generated using forward and reverse ssDNA oligos (IDT). They were designed to only contain one spacer and PAM of interest. All other NGG sequences outside of the spacer and PAM were altered to NTG. The oligo pairs used are shown in Supplementary File 2. The pairs were diluted to 1 mM in Duplex Buffer (IDT), and then annealed by first mixing at a 1:1 ratio (20 μl 1 mM forward primer, 20 μl 1 mM reverse primer, and 4 μl 1M NaCl). The mixture was heated to 95°C for 5 min and cooled via thermocycler at -0.6°C per minute until reaching room temperature (20°C, amounting to ∼2.5 h). The annealed DNA samples were dialyzed in ddH_2_O and 100 μg of dsDNA was electroporated into competent RN4220 pGG32-ΔtrL cells, as described previously (35). Cells were recovered for 2h in BHI at 37°C with agitation.

Plasmids from the transformed staphylococci were isolated via the miniprep protocol described above. The CRISPR array was then PCR amplified using primers oNA169 and oNA170 (which are highly sensitive for the repeat sequence), and the PCR reactions were purified via the QIAquick PCR Purification Kit, following its corresponding protocol. To maximize yield, the amplified CRISPR array was isolated by automated gel electrophoresis using a PippinHT machine (model HTG-3010), following the manufacture's PippinHT Quick Guide protocol. For precise DNA size selection, a PippnHT 3% cassette was used with a timed protocol set for sample extraction between 26 and 35 min (34). The eluates extracted from the PippinHT cassette were then sequenced on an Illumina MiSeq, as described above. Sequence analysis was done in Python to determine the number of spacers acquired from the NNGG, vs the NGG PAMs, on the transformed dsDNA oligos (see above description).

### Statistics and reproducibility

All experiments were independently reproduced three times, unless otherwise stated. Statistical tests were carried out in GraphPad Prism 9.4.1. No statistical methods were used to predetermine sample size.

## RESULTS

### Spacers targeting phage sequences flanked by NAGG PAMs can mediate type II-A CRISPR-cas immunity

**Figure 1.**
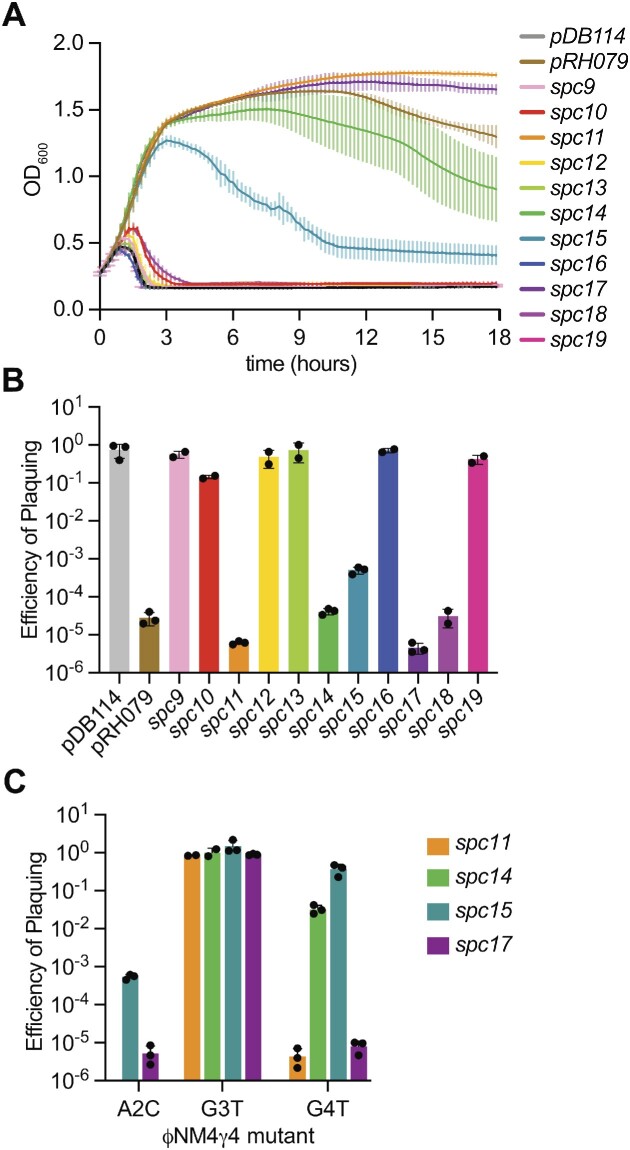
Spacers targeting phage sequences flanked by NAGG PAMs can mediate type II-A CRISPR-Cas immunity. (**A**) Cell survival, measured as the OD_600_ values after ФNM4γ4 infection of cultures carrying plasmids harboring the type II-A CRISPR-Cas locus of *S. pyogenes*, programmed with different spacers that target protospacers flanked by non-canonical PAMs, as well as a non-targeting control (pDB114) and a plasmid with a canonical spacer (pRH079). The average curves of three different replicates are shown, with ± standard deviation (StDev) values shown in lighter colors. (**B**) Propagation ability of ΦNM4γ4 on staphylococci harboring the different plasmids described in (A), measured as efficiency of plaquing (EOP). Mean ± StDev values of three independent experiments are shown. (**C**) Same as in (B) but using cultures harboring spacers 11, 14, 15 or 17, and infecting with mutant phages with substitutions in different nucleotides of the NAGG PAM sequence.

Previous work from our lab measured spacer acquisition by a *S. pyogenes* type II-A CRISPR-Cas system (Supplementary Figure S1A). This locus was cloned into the chloramphenicol-resistant staphylococcal vector pC194 (30) to generate plasmid pWJ40 (31) and introduced into *S. aureus* RN4220 (29). Bacterial cultures were infected with the lytic staphylococcal phage ФNM4γ4 (17), and next generation sequencing was used to determine the sequence and frequency of the phage DNA inserted into the CRISPR array (17). In these studies, we detected a low frequency of reads for spacers that match regions of the phage not flanked by the NGG PAM (17). In order to evaluate whether these sequences correspond to functional spacers, we evaluated their ability to provide immunity to the bacterial host. We decided to study sequences that displayed at least 10 reads (Supplementary Table S1). First, we confirmed the absence of a canonical PAM through Sanger sequencing of the ФNM4γ4 targets (data not shown). Next, we cloned each spacer into pDB114, a plasmid that lacks the *cas1*, *cas2*, and *csn2* genes involved in spacer acquisition and contains a modified CRISPR array with a single engineered spacer (36) (Supplementary Figure S1B). Cultures of staphylococci harboring the different plasmids were then infected with ФNM4γ4 at a multiplicity of infection (MOI) of 1, and their growth was followed by monitoring optical density at 600 nm (OD_600_) for 18 h (Figure 1A). We found that spacers 11 and 17 provided full protection (similar to our positive control, pRH079, a plasmid derived from pDB114 that harbors a functional, canonical spacer (17)), and spacers 14 and 15 provided partial protection. We also tested the set of spacers for their ability to limit phage propagation by seeding ФNM4γ4 on lawns of staphylococci harboring pDB114 plasmids programmed with different spacers (Supplementary Figure S1C). The plaque forming units (PFU) were enumerated to calculate the efficiency of plaquing (EOP) for each spacer, relative to the PFUs obtained on lawns of bacteria harboring pDB114, without a targeting spacer (Figure 1B). Consistent with our observations of bacterial growth, the presence of spacers 11 and 17 dramatically reduced the EOP by five orders of magnitude, and spacers 14 and 15 by approximately 3–4 orders of magnitude. Interestingly, spacer 18, which did not protect bacterial cultures (Figure 1A), was able to limit phage production by approximately 4 orders of magnitude in the plaque assay.

We looked for sequence similarities in the PAM region of the targets of spacers 11, 14, 15, 17 and 18 and found a conserved NAGG motif (Supplementary Table S1). In order to determine whether this motif is required for immunity and if so, which of these nucleotides (A2, G3 or G4 of the N1-A2-G3-G4 sequence) is important for target recognition by Cas9, we isolated phages that can escape the immunity mediated by spacers 11, 14, 15 and 17 from our plaque assays (Supplementary Figure S1C) and sequenced the target region. We found that escapers harbored mutations in either the seed or PAM regions of the target that are known to be detrimental for efficient Cas9 cleavage and immunity against ФNM4γ4 (22). Interestingly, in all cases, PAM mutations were observed only in G3 (Supplementary File 1). To corroborate this finding, we measured the EOP of selected escapers containing a T nucleotide in this position of the motif and, expectedly, we obtained values close to 1; i.e. their respective spacers were not able to provide immunity (Figure 1C). Since we were unable to isolate phages carrying mutations in A2 or G4, we decided to engineer substitutions of these nucleotides, A2C and G4T. NAGT motifs had a detrimental effect in the targeting of the ‘weaker’ spacers (14) and (15), but not on the ‘stronger’ spacers 11 and 17 (Figure 1C). NCGG motifs flanking the *spc15* and *spc17* targets, on the other hand, were still able to support Cas9 targeting (Figure 1C). Unfortunately, we were unable to mutate A2 in the targets of spacers 11 and 14 (most likely the mutant phages are not viable). Altogether, these data demonstrate spacers matching protospacers followed by NAGG sequences (heretofore called ‘NAGG spacers’) can be acquired during the type II-A CRISPR-Cas response and that these NAGG sequences constitute a non-canonical PAM that can support efficient Cas9-mediated phage defense, with the central G3 nucleotide being essential for immunity.

### Cas9 can efficiently cleave DNA targets followed by a non-canonical NAGG PAM

**Figure 2.**
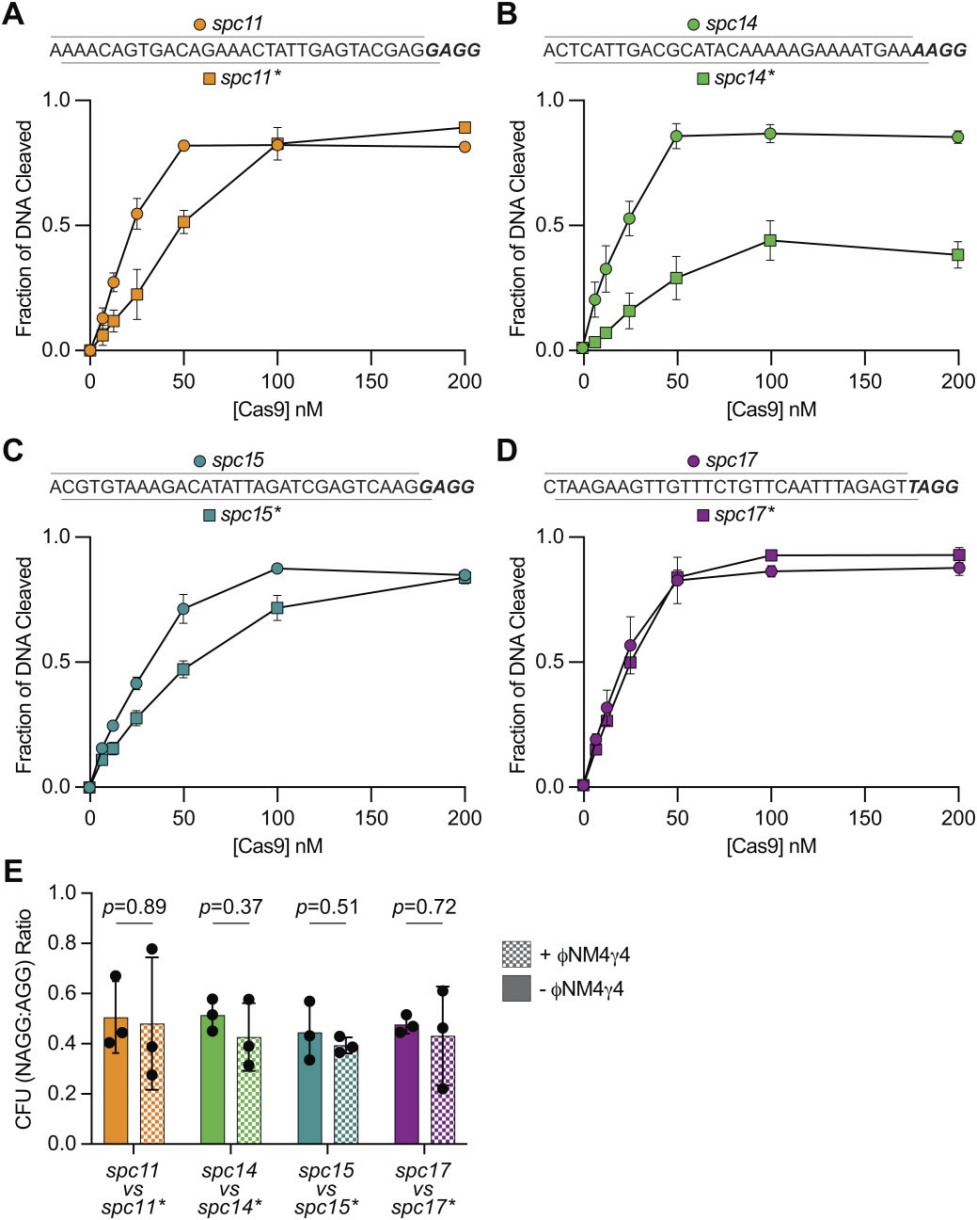
Cas9 can efficiently cleave DNA targets followed by a non-canonical NAGG PAM. (**A**) *In vitro* cleavage assay of a ∼1 kb PCR product containing the *spc11* and spc11* target DNA sequences, incubated with increasing concentrations of a 1:1:1 mix of Cas9:tracrRNA:crRNA: 0, 6.25, 12.5, 25, 50, 100 and 200 nM. Substrates and cleavage products were separated and quantified using a Tapestation bioanalyzer. Mean ± StDev values of three independent experiments are shown. (**B**) Same as (A) but testing *spc14* and spc14* target and crRNA sequences. (**C**) Same as (A) but testing *spc15* and spc15* target and crRNA sequences. (**D**) Same as (A) but testing *spc17* and spc17* target DNA and crRNA sequences. (**E**) Growth competition of staphylococci carrying pDB114 harboring an NAGG spacer or its corresponding canonical AGG spacer and different antibiotic resistance cassettes (chloramphenicol-resistant for NAGG spacers, kanamycin- and chloramphenicol-resistant for AGG spacers), mixed at a 1:1 ratio in the absence of antibiotics and infected with ΦNM4γ4 for 24 h. For each NAGG-AGG spacer pair, the ratio of CFU of the NAGG strain to the AGG strain was determined at 0 and 24 h by plating on selective agar plates. Mean ± StDev values of three independent experiments are shown. *P* values obtained by a Student's *t*-test are shown.

To further investigate the immunity mediated by NAGG spacers, we evaluated the ability of Cas9 to cleave their targets, *in vitro*. As positive controls we used the canonical spacers for these targets; i.e. we shifted the spacer sequence one nucleotide to include the ‘N’ in the protospacer, and thus have a canonical AGG PAM (Figure 2A–D). We indicated this shift with an asterisk (for example *spc11* targets the NAGG PAM; spc11* targets the AGG PAM). These spacers mediated full immunity *in vivo* (Supplementary Figure S2A). We then designed crRNAs corresponding to those originated by each of the different spacers (see Supplementary File 2 for sequences) and associated them with Cas9 and tracrRNA to obtain ribonucleoprotein complexes. The target phage DNA corresponding to each NAGG spacer and AGG spacer pair (1 kb, with the protospacer sequence in the middle) was amplified via PCR, purified, and used as a substrate for the Cas9 complex. DNA cleavage at increasing concentrations of the nuclease was quantified using a Tapestation bioanalyzer (Supplementary Figure S2B–E) and plotted (Figure 2A-D). We found that with the exception of *spc14* crRNA, which mediated ∼ 50% of the cleavage mediated by the spc14* crRNA, all other crRNAs targeting an NAGG PAM substrate conveyed similar cleavage as their counterpart guides that direct AGG PAM recognition. These results suggest that Cas9 can recognize and cleave targets harboring an NAGG non-canonical PAM.

### NAGG-spacers mediate an immune response comparable to that of canonical spacers

The above results suggest that NAGG-spacers are able to support considerable levels of both immunity and Cas9 cleavage. In order to assess their phage targeting efficiency more accurately, we directly compared them with their canonical counterparts in a competition experiment. To this end, we introduced a kanamycin-resistant cassette into the *S. aureus* RN4220 genome to use as host for the pDB114 plasmid harboring spacer 11*, 14*, 15* or 17*. The resulting AGG spacer strains and their corresponding NAGG spacer strains (kanamycin and chloramphenicol resistant, and only chloramphenicol resistant, respectively) were mixed at a 1:1 ratio and the cultures were infected with ФNM4γ4. After 24 h of growth, samples were plated on agar containing either chloramphenicol only or kanamycin and chloramphenicol, to enumerate colony forming units (CFU) and calculate the NAGG/AGG ratio for each spacer pair. Mixed cultures that were not infected with phage were used as controls. For all four spacers, we found that that this ratio did not significantly change from 0.5 (Figure 2E), a result that suggests that the NAGG spacers provide similar immunity as the canonical ones.

### Non-acquired NAGG spacers provide efficient immunity

**Figure 3.**
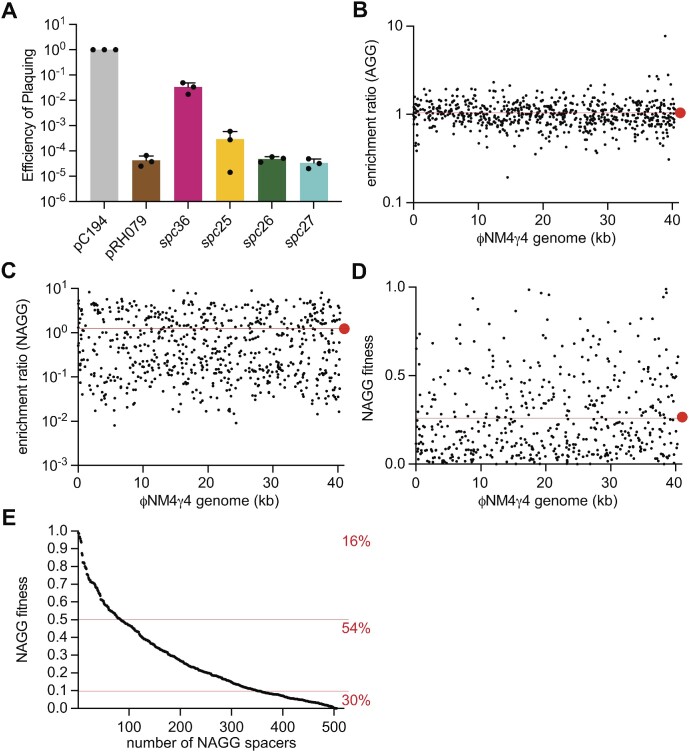
Non-acquired NAGG spacers provide efficient immunity. (**A**) Propagation ability of ΦNM4γ4 on staphylococci carrying plasmids harboring the type II-A CRISPR-Cas locus of *S. pyogenes*, programmed with different spacers that target protospacers flanked by non-canonical PAMs, as well as a non-targeting control (pC194) and a plasmid with a canonical spacer (pRH079), measured as efficiency of plaquing (EOP). Mean ± StDev values of three independent experiments are shown. (**B**) Enrichment ratios of canonical spacers targeting protospacers flanked by an AGG PAM, calculated using NGS data as the frequency of reads of each spacer sequence after ΦNM4γ4 infection, relative to that spacer's frequency value without phage infection. Each dot represents a different spacer sequence, plotted at its location within the ΦNM4γ4 genome. The red line and dot represent the average enrichment ratio, 1.05. Standard deviation: ±0.43. (**C**) Same as (B) but for NAGG spacers. Average ratio: 1.23; standard deviation: ±1.57. (**D**) Fitness of NAGG spacers compared to AGG spacers, in a competition assay of the NAGG and AGG spacer libraries, mixed at a 1:1 ratio and infected with ФNM4γ4. Fitness is calculated as the frequency of each NAGG spacer relative to the total frequency of the NAGG and its cognate AGG spacer combined. Each dot represents the fitness of a different NAGG spacer sequence, plotted at its location within the ΦNM4γ4 genome. The red line and dot represent the average fitness, 0.27. (**E**) Fitness values described in (D) plotted in highest to lowest order. Red lines separate NAGG spacers with fitness values higher than 0.5, between 0.5 and 0.1, and less than 0.1, and the percentage of NAGG spacers falling between each range is noted.

Given that the four NAGG spacers that we identified in spacer acquisition assays are able to provide substantial immunity, we decided to investigate four NAGG spacers that were not detected in our previous spacer acquisition experiments. We chose spacers for which the adjacent canonical spacer (which directs Cas9 to an AGG PAM) displayed either a low (spacers 36 and 25) or a high (spacers 26 and 27) acquisition frequency (Supplementary Table S1). We found that, while *spc36* mediated a low, yet significant, level of defense (a reduction in EOP of two orders of magnitude), spacers 25, 26 and 27 provided an immune response as strong as a control spacer targeting a canonical AGG PAM (Figure 3A and Supplementary Figure S3A). This result suggests that many of the NAGG spacers that are not detected in our spacer acquisition assays can convey efficient immunity. To test this hypothesis thoroughly, we built a spacer library into pDB114 plasmids, targeting all 787 NAGG sequences present in the ФNM4γ4 genome. We also constructed a library of the same 787 spacer sequences shifted by one nucleotide, thus targeting protospacers followed by AGG PAMs. Plasmids harboring each library were extracted from staphylococcal cultures, the spacer region was amplified via PCR, and the products were subjected to next generation sequencing (NGS) to confirm that all spacer sequences were present at similar levels (Supplementary File 1). We then grew each culture for 24 h, in the presence or absence of ФNM4γ4 and used NGS to evaluate the spacer content within the surviving population. We obtained reads in all four samples for 680 of the 787 spacer sequences, and used the data to calculate an enrichment ratio, defined as the read frequency after phage infection relative to the frequency obtained for the uninfected culture at the end of the experiment (Supplementary File 1). For the AGG spacers, the ratios were clustered around 1 (average 1.05), and therefore the standard deviation was low, ±0.43 (Figure 3B). On the other hand, NAGG spacers displayed more irregular values. Enrichment ratios ranged from 10 to 0.1 (average 1.23), with an overall higher standard deviation of ±1.57 (Figure 3C). These results show that while canonical spacers are similarly effective in their ability to provide defense, NAGG spacers mediate more variable targeting efficiency.

We also used the NGS data to investigate the importance of different nucleotides in the first position of NAGG PAMs for targeting by Cas9. We compared the mean of the enrichment ratios obtained for AAGG, CAGG, GAGG and TAGG sequences with the overall mean (Supplementary Table S2 and Supplementary File 1). Interestingly, we found a highly significant preference for A and disfavor for C nucleotides in this position. Also, it was noted that in protospacers 11, 14, 15 and 17, the last base of the seed sequence is identical to the N of the NAGG PAM (Figure 2A–D). To investigate how this combination of identical nucleotides contributes to Cas9 immunity, we first looked at the importance of the last nucleotide of the seed sequence using our NGS data, as explained above. In this case, we did not find a highly significant statistical difference for the mean targeting enrichment values of any last nucleotide of the seed, when compared to the overall mean (Supplementary Table S2 and Supplementary File 1). We then analyzed the data for the dinucleotide sequences composed of the last base of the seed sequence and the N of the NAGG PAM. We found a highly significant statistical difference for the average targeting of AA combinations, but not for CC, GG nor TT (Supplementary Table S2 and Supplementary File 1). Given the substantial preference of the second of these two A nucleotides (the first A in an AAGG PAM, see above), we believe these results reflect this bias but do not indicate that AA in the *spc14* target (nor GG in the *spc11* and *spc15* targets, nor TT in the *spc17* target) seed-PAM dinucleotides are more efficient for Cas9 targeting, at least in our assays.

To compare more accurately the immunity provided by canonical and NAGG spacers, we performed a competition experiment in which both libraries were combined at a 1:1 ratio, and the resulting mixed culture was infected with ФNM4γ4 phage for 24 h. This experimental set up resembles, to some extent, the spacer acquisition process, in which many diverse spacer sequences are inserted into the CRISPR array of different individual bacteria during phage infection. Using NGS, we first confirmed that the different spacers in this culture were similarly represented. 747 spacer pairs of the 787 total cloned were found in the library, with the great majority present at frequencies ranging from 10^−4^ to 10^−3^, for both canonical and NAGG spacers (Supplementary Figure S3B-C and Supplementary File 1). We then calculated the enrichment ratio as before, comparing the spacer reads obtained from infected and non-infected cultures after 24 h of growth (Supplementary File 1). Of the 747 original NAGG-AGG spacer pairs, 505 spacers appeared in all four experiments. We found that the enrichment ratios for the canonical spacers clustered around values of 10 or 0.1 (Supplementary Figure S3D), suggesting the presence of a subfraction of spacers that provide better immunity in these conditions. The enrichment ratios for the NAGG spacers also seemed to be separated into values either higher or lower than 1, but displayed a more scattered distribution (Supplementary Figure S3E). More importantly, in this experiment the immunity mediated by each NAGG spacer can be directly compared to the internal control provided by its corresponding canonical, AGG spacer. Therefore, we were able to calculate the fitness of each NAGG spacer as its enrichment ratio relative to the sum of the AGG and NAGG spacer enrichment ratios (Supplementary File 1). Fitness values ranged between 1 and 10^−4^ all across the phage genome, with an average of 0.27 (Figure 3D). On one hand, 16% of the NAGG spacers displayed a fitness better than their canonical counterparts (>0.5); on the other hand, only 30% showed a fitness value of less than 0.1 (Figure 3E). Therefore, we conclude that while most AGG spacers outperform their corresponding NAGG, they do so by less than an order of magnitude. More generally, the fact that NAGG spacers are not dramatically outcompeted by canonical ones in a mixed population suggests that these spacers, even if not acquired by the type II-A CRISPR-Cas system, are able to mount an efficient anti-phage defense.

### Strong bias against the acquisition of NAGG spacers during the type II-A CRISPR immunization stage

**Figure 4.**
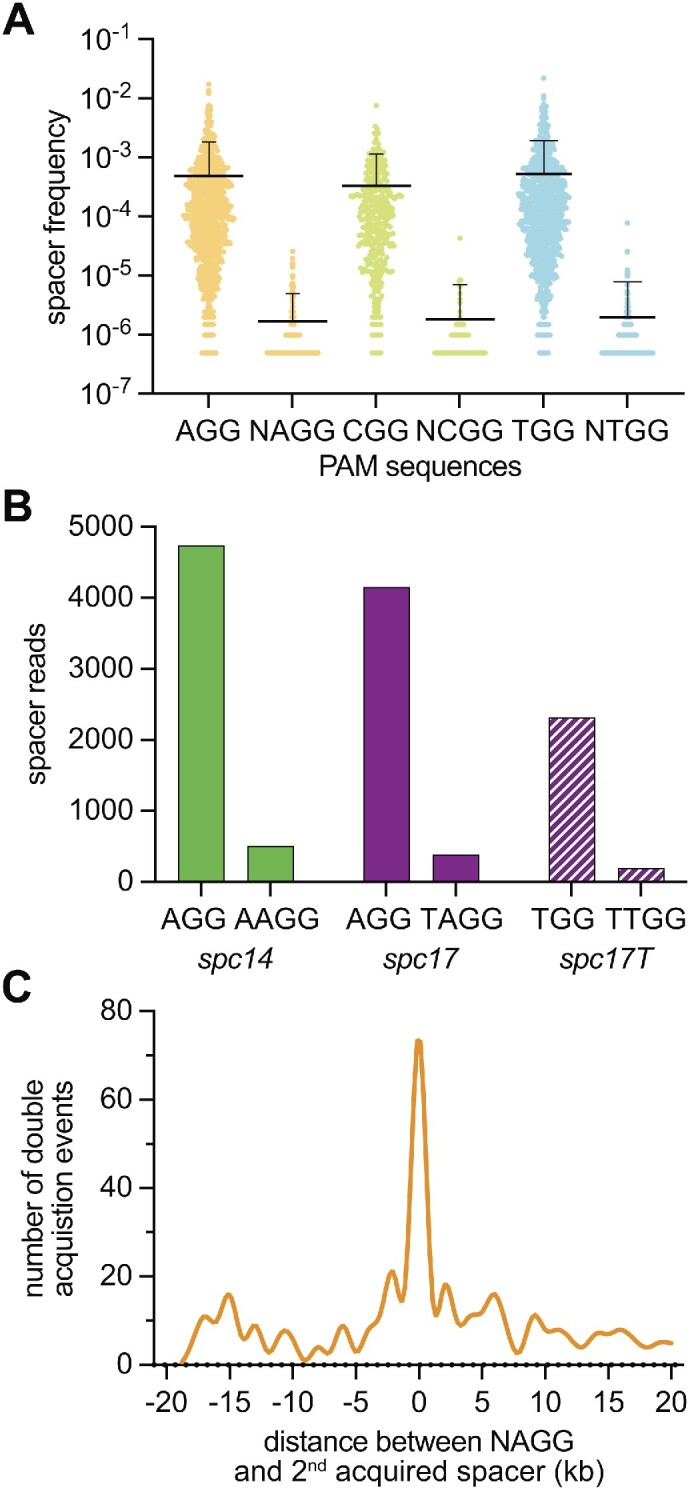
NAGG spacers are rarely acquired during the type II-A CRISPR immune response. (**A**) Scatter plot of frequency of reads for spacers matching targets containing different PAM sequences, obtained via NGS analysis of spacer acquisition after transformation of sonicated ФNM4γ4 DNA. Mean ± StDev is shown. (**B**) Number of spacer reads, detected by NGS of staphylococcal cultures carrying pGG32-ΔtrL, transformed with oligonucleotides harboring PAMs and pre-spacer sequences corresponding to different spacers. (**C**) Distance between the targets in the ΦNM4γ4 genome, specified by the first and second spacers acquired after infection of naïve staphylococci carrying the type II-A CRISPR-Cas system of *S. pyogenes*, with the first spacer matching a target with an NAGG PAM. The position of the target of the first NAGG spacer on the ΦNM4γ4 genome is set to 0 kb, and each 1 kb bin contains the number of second spacers acquired from targets that given genomic distance from the first target on the phage DNA.

Our previous results showed that NAGG spacers can mediate substantial, if not equal, immunity as spacers matching targets flanked by canonical PAMs. In principle, if these spacers are acquired, they should provide sufficient immunity to be maintained within the bacterial population. Since this is not the case, we hypothesized that their relative absence from the spacer repertoire could be due to a low efficiency of acquisition. To test our hypothesis, we looked at the frequency of acquisition of NAGG spacers in the absence of phage infection. This eliminates changes in spacer frequency that are due to the variable ability of each spacer to defend and promote the growth of the host. We looked at spacer acquisition data previously generated in our lab after transformation (via electroporation) of staphylococci with ФNM4γ4 phage DNA, sheared into ∼150 bp fragments by sonication (35). We compared the frequencies of acquisition of AGG and NAGG spacers and found that, on average, canonical spacers were integrated 2–3 orders of magnitude more than NAGG spacers (Figure 4A and Supplementary File 1). Very similar results were obtained when we compared CGG/NCGG and TGG/NTGG spacers (GGG and NGGG cannot be compared because both sequences are canonical PAMs), not only in the difference between averages, but also in their absolute values.

To further test these results, we evaluated the acquisition of double-stranded DNA oligonucleotides containing either the sequence of *spc14* and its PAM, or *spc17* and its PAM, following transformation via electroporation (Figure 4B). These sequences contain a single GG dinucleotide that can be recognized by Cas9 (17,23) (see Supplementary File 2 for their full sequences). The total number of reads for acquired canonical spacers was approximately one order of magnitude higher than the number of reads for NAGG spacers. We also made an A to T modification in the oligonucleotide sequence harboring *spc17*, to generate a TGG PAM. Although acquisition for the canonical TGG spacer was lower than that of the AGG spacers, acquisition of the NTGG spacer was similarly low to that of the unmodified *spc17* oligonucleotide (Figure 4B). While the trend of these results is consistent with the acquisition of spacers from fragmented phage DNA, the difference in the acquisition of the sequence upstream of the AGG compared to that of the NAGG motif, is much less than what is observed in Figure 4A. We believe that this is a consequence of the presence of only two main protospacer sequences in the oligonucleotide substrate, in contrast to the 2687 NGG sequences and 2687 NNGG sequences in the ΦNM4γ4 genome. All of these viral sequences are competing for the spacer acquisition machinery and it is conceivable that in such extreme conditions a ∼10× difference in acquisition efficiency between an NGG spacer and its NNGG counterpart is amplified to ∼1000×. Therefore, we conclude that these data demonstrate a strong bias against the acquisition of spacers derived from non-canonical PAM sequences, even when those sequences can support efficient DNA targeting and cleavage.

### NAGG spacers enable primed spacer acquisition

Interestingly, NAGG spacers represent a form of ‘slipped’ spacer that was previously described in type I CRISPR-Cas systems (37). In the case of the NAGG PAM, the spacer has a ‘+1 slip’ where the acquired spacer matches a target in which the PAM is not immediately adjacent to the protospacer but instead is one nucleotide away from it (37). Analysis of these spacers revealed that, as opposed to the NAGG spacers, they do not provide efficient immunity. Instead, they can stimulate primed CRISPR adaptation, i.e. the acquisition of additional spacer sequences from invaders (38). Type II-A systems use free DNA ends as substrates for spacer acquisition (20,21), therefore the presence of pre-existing spacers that mediate Cas9 cleavage of the viral DNA enhances the capture of phage sequences from the vicinity of the cut site (22). As a result of this priming mechanism, during the events of double spacer acquisition, the second spacer integrated into the CRISPR array targets a location of the genome that is close to the region targeted from the first acquired spacer (22,39).

To investigate if NAGG spacers are slipped spacers that can mediate priming, we looked for double spacer acquisition events in a set of spacers acquired after ФNM4γ4 infection (40). We found 405 unique events in which the first acquired sequence matched a protospacer flanked by an NAGG PAM in the phage genome, and calculated the distance between the first and second acquired spacers, when mapped onto the ФNM4γ4 genome (Supplementary File 1). Consistent with previous similar analysis for NGG spacers (22,39), the histogram of these distances revealed that the majority of the second spacers map within 1 kb of the protospacer targeted by the NAGG spacer (Figure 4C). This spacer distribution strongly suggests that NAGG spacers enable priming and therefore represent an example of slipped spacers that can also mediate targeting.

## DISCUSSION

Here, we investigated viral sequences that are rarely acquired as spacers during the type II-A CRISPR-Cas response against phage infection. Many of these sequences result in the recognition of viral targets that contain a non-canonical PAM of the sequence NAGG. We found that these spacers can mediate substantial immunity, even those with sequences that are not acquired at all. In contrast to their ability to participate in effective targeting, the acquisition rate of NAGG spacers is very low. Therefore, we conclude that the failure to acquire these sequences, and not their efficiency in promoting phage defense, results in their low abundance within bacterial populations. Our findings highlight a mechanistic separation of the role of Cas9 in PAM recognition during spacer acquisition (17) and DNA cleavage (23). This is reminiscent of the findings for type I systems, which display flexible requirements for PAM recognition during DNA targeting, with many sequences supporting efficient DNA cleavage, but a much more restricted repertoire of PAM sequences for acquired spacers (41). This finding has led to the proposition of using the term Spacer Acquisition Motif (SAM) when referring to the sequence recognized by the acquisition machinery and the term Target Interference Motif (TIM) when referring to the sequence recognized by crRNA-guided nuclease complexes (42). Previous work studying the type II-A system of *Streptococcus thermophilus* also identified a minority of spacers carrying imperfect PAMs harboring 1–2 nucleotides that differ from the canonical AGAAW motif (43). The authors speculated that the existence of non-canonical spacers may reflect the targeting flexibility of Cas9. Our work suggests that, similarly to the NAGG spacers present in the *S. pyogenes* type II-A system, the non-canonical spacers captured by the *S. thermophilus* system may indeed be functional, yet likely under-sampled during the spacer acquisition stage.

While high PAM recognition flexibility during DNA cleavage would enable the targeting of escaper phages containing mutations in this motif, it is more difficult to envision the evolutionary forces behind stringent PAM recognition during spacer acquisition. Possibly, this is observed as a consequence of the extremely low frequency of spacer integration, combined with the variable targeting efficiency of the NAGG spacers. It is believed that CRISPR systems have evolved to have an extremely low rate of spacer acquisition to limit ‘autoimmunity’; i.e. the integration of genomic sequences into the array that will direct Cas9 to the bacterial chromosome (21,44). Such low frequency implies that the survival of the bacterial population depends on the occurrence of relatively few spacer acquisition events. In this context, the acquisition of spacers that do not always guarantee efficient targeting, as is the case for NAGG spacers, would have favored the evolution of a strict selection of NGG spacers by the acquisition machinery, in order to ensure that the small population of adapted cells is endowed with a fully competent set of spacers.

We also found that NAGG spacers can enhance further spacer acquisition. This result corroborates the ability of these non-canonical spacers to mediate efficient DNA cleavage, since type II-A systems use free DNA ends as substrates for new spacers (20–22) and therefore cleavage of the viral DNA by Cas9 generates these substrates and stimulates spacer acquisition from the target site. This phenomenon, also known as priming (38), has similarities in type I CRISPR systems, particularly to the priming mediated by ‘slipped’ spacers (37). Slipped spacers originate from imprecise acquisition events and match a protospacer in which the PAM is shifted one nucleotide. Given that the targets of these spacers lack an optimal PAM, they mediate inefficient DNA cleavage, but are able to stimulate primed spacer acquisition in type I CRISPR systems. In this context, NAGG spacers constitute slipped spacers for the type II-A CRISPR system of *S. pyogenes* that enhance further spacer acquisition but can also mediate efficient DNA cleavage.

Structural studies showed that *S. pyogenes* Cas9 contains two arginines, each of which interacts with one of the guanine residues of the canonical NGG target motif (23). We currently do not know how these residues are also able to recognize NAGG sequences—whether they interact with A2 and G3, or with G3 and G4, or whether they adopt a different recognition conformation. However, given that Cas9 participates in spacer acquisition as part of a ‘supercomplex’ that contains also Cas1, Cas2 and Csn2 (17,19), it is tempting to speculate that PAM recognition is more stringent in this potentially confined context, than during target DNA cleavage, which is performed by Cas9 alone (7).

Our results are in line with, and also expand upon earlier work, regarding both spacer acquisition and DNA targeting by the *S. pyogenes* type II-A CRISPR-Cas system. In a previous study that investigated the distribution of spacers within bacterial communities carrying this system, it was determined that the spacer patterns are established early during the CRISPR-Cas immune response and correlate with spacer acquisition rates, but not with spacer targeting efficiency (35). Here we show that this is also the case for non-canonical NAGG spacers, which are poorly acquired despite their relatively high efficiency of targeting, and therefore remain rare within the host population. With relation to Cas9 targeting, previous comprehensive analysis of all the possible 5-nucleotide sequences flanking the 3' end of the protospacer in the *S. pyogenes* type II-A system, found that both NAG and NNGG motifs can support a low efficiency of targeting (∼50% and 20%, respectively) (45–47). Our evaluation of the effect of different mutations of the NAGG sequence showed that changes in G3, which eliminate both the NAG and NNGG motifs, are the most detrimental for the defense mediated by NAGG spacers (Figure 1C). We also found that A2C substitutions have a substantial effect on immunity (Figure 1C) and that a target flanked by the non-canonical PAM sequence TTGG failed to mediate defense (Figure 1B-C). These results indicate that, while NAGG PAMs enable Cas9 target recognition and cleavage, this is not the case for NCGG and NTGG motifs. Finally, the effects of the G4T mutations (NAGT PAM) varied with different protospacer sequences. Therefore, we believe that this is the least critical position of the NAGG PAM, a result that correlates with the higher targeting efficiency previously observed for NAG spacers, compared to NNGG spacers (see above).

Our study focused on *S. pyogenes* Cas9, and therefore it remains to be determined whether other type II CRISPR-Cas systems can also recognize targets flanked by sequences that deviate from canonical PAMs. On the other hand, given that the great majority of CRISPR-based gene editing applications rely on *S. pyogenes* Cas9 (25,26), our findings have implications for these technologies. In particular with regards to the off-target effects of gene editing (48), understanding the flexibility in target recognition by Cas9 could help to identify additional off-target sites and decrease the possibility of collateral cleavage of the human genome.

## Supplementary Material

gkad501_Supplemental_Files

## Data Availability

The spacer sequences and accompanying statistics that are discussed in this paper are provided in Supplementary File 1. The original FASTQ files from the NGS experiments have been uploaded to the SRA database [accession # PRJNA972507]. The raw data from this study are available from the corresponding author upon request. Custom python scripts are deposited at Figshare: https://figshare.com/articles/software/kenneyc_etal_code_2023/22970996.
