# Supplementary material for: Rarely acquired type II-A CRISPR-Cas spacers mediate anti-viral immunity through the targeting of a non-canonical PAM sequence
Source: Nucleic Acids Res. 2023 Jun 9;51(14):7438–50. doi: 10.1093/nar/gkad501 (PMC10415147; doi:10.1093/nar/gkad501)
Supplement: gkad501_Supplemental_Files [file gkad501_supplemental_files.zip › Kenney_Suppl_Mat.pdf]

Rarely acquired type II-A CRISPR-Cas spacers mediate anti-viral immunity through the targeting of a non-canonical PAM sequence

Claire T. Kenney[1] and Luciano A. Marraffini[1,2,*,#]

[1]Laboratory of Bacteriology, The Rockefeller University, 1230 York Ave, New York, NY 10065, USA.

[2]Howard Hughes Medical Institute, The Rockefeller University, 1230 York Ave, New York, NY 10065, USA.

*Correspondence to: marraffini@rockefeller.edu

SUPPLEMENTARY FIGURES

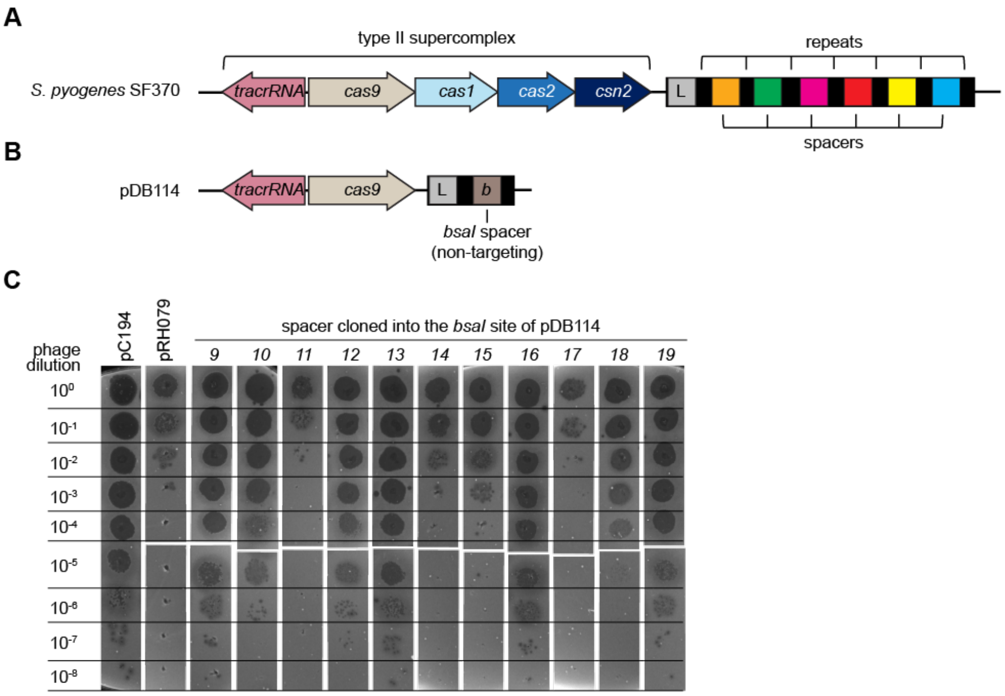

Supplementary Figure S1. Design of a two-plasmid system for the study of the effect of pre-existing spacers in type II-A CRISPR-Cas spacer acquisition. (**A**) Type II-A CRISPR-*cas* locus of *S. pyogenes* SF370. Black rectangles, CRISPR repeats; colored and numbered rectangles, spacers; "L", leader sequence; the genes encoding the type II-A supercomplex are noted. (**B**) pDB114 construct. The *tracrRNA*, *cas9* and two repeats from the *S. pyogenes* SF370 type II-A system were cloned into the staphylococcal vector pC194 containing a chloramphenicol resistance gene. *bsaI* restriction sites were incorporated in-between the repeats for the cloning of spacers using annealed oligonucleotides. (**C**) Detection of plaque formation of lawns of staphylococci harboring pDB114 plasmids with different spacers and pC194 or pRH179 as controls for the lack or presence of type II-A CRISPR immunity, respectively. A representative image of three independent experiments is shown.

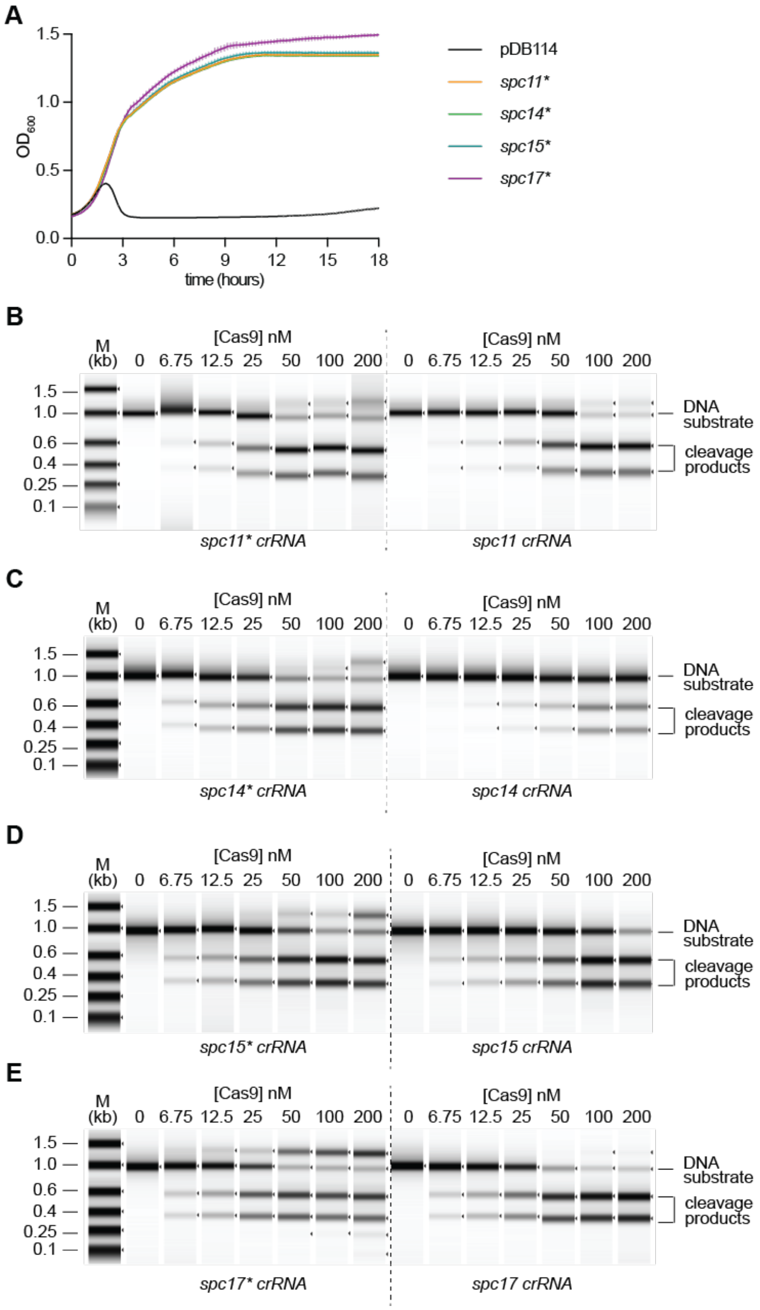

Supplementary Figure S2. ***In vitro*** **Cas9 DNA cleaving assays.** (**A**) Cell survival measured as the OD_{600} values after ΦNM4ɣ4 infection of cultures carrying pDB114 programmed with different spacers that target protospacers flanked by canonical PAMs, as well as a non-targeting control (pDB114). The average curves of three different replicates are shown, with +/- StDev values shown in lighter colors. (**B**) *In vitro* cleavage assay of a ~ 1kb PCR product containing the *spc11* and *spc11** target DNA sequences, incubated with increasing concentrations of a 1:1:1 mix of Cas9:tracrRNA:crRNA: 0, 6.25, 12.5, 25, 50, 100 and 200 nM. The crRNA sequence contained either the *spc11* or *spc11** sequence. Substrates and cleavage products were separated and visualized using a Tapestation bioanalyzer. A representative image of three independent

experiments is shown. (**C**) Same as (**B**) but testing *spc14* and *spc14** target DNA and crRNA sequences. (**D**) Same as (**B**) but testing *spc15* and *spc15** target DNA and crRNA sequences. (**E**) Same as (**B**) but testing *spc17* and *spc17** target DNA and crRNA sequences.

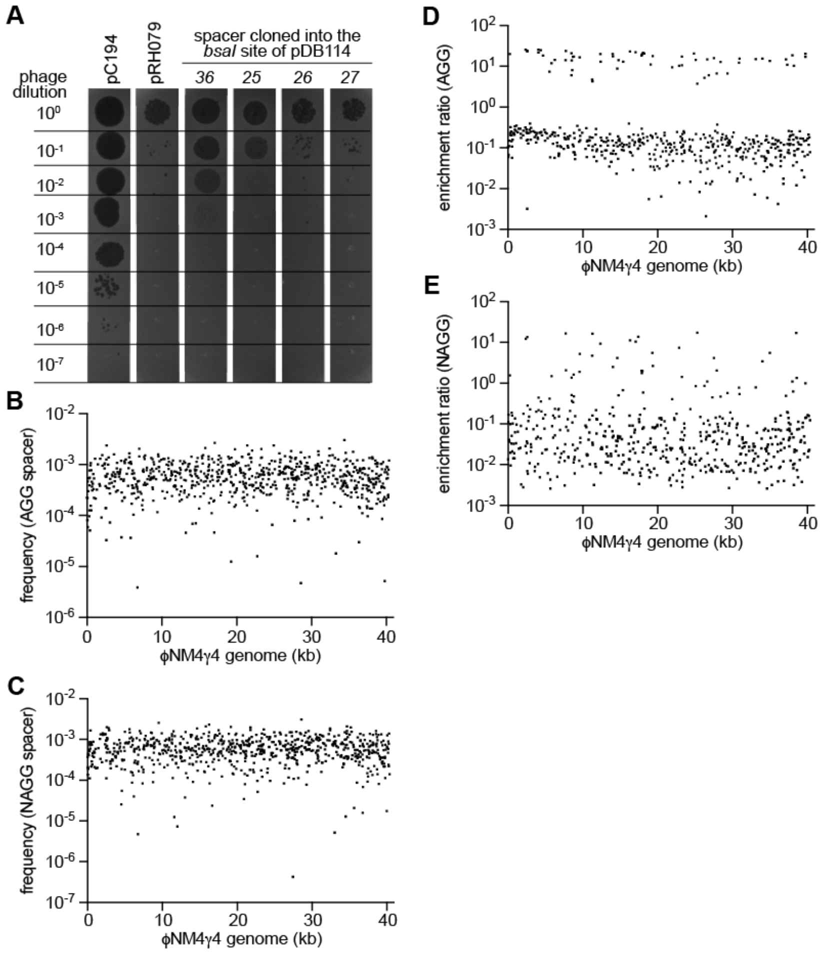

Supplementary Figure S3. Enrichment of AGG and NAGG spacers within spacer libraries infected with ΦNM4ɣ4. (**A**) Detection of plaque formation of lawns of staphylococci harboring pDB114 plasmids with different spacers and pC194 or pRH179 as controls for the lack or presence of type II-A CRISPR immunity, respectively. A representative image of three independent experiments is shown. (**B**) Frequency of each AGG spacer sequence in the library. Each dot represents a different spacer sequence, plotted at its location within the ΦNM4ɣ4 genome. (**C**) Same as (**B**) but for the NAGG spacer library. (**D**) Enrichment ratio of canonical spacers targeting protospacers flanked by an AGG PAM, calculated using NGS data as the frequency of each spacer sequence after ΦNM4ɣ4 infection of cultures having both AGG and NAGG spacer libraries mixed at a 1:1 ratio, relative to the frequency value without infection. Each dot represents a different spacer sequence, plotted at its location within the ΦNM4ɣ4 genome. (**E**) Same as (**D**) but for NAGG spacers.

SUPPLEMENTARY TABLES

Supplementary Table 1. Spacers investigated in this study

Name	Sequence (5’-3’)	PAM[a]	freq-1[b]	freq-2[c]
spc9	GCATGTTATAATCCACACCCTTGCGTTTAA	TAAG	0	3.91E-06
spc10	TCGAATAACTCACGTTCCATTGAATACTGT	GTAG	0	1.73E-05
spc11	AAAACAGTGACAGAAACTATTGAGTACGAG	G**AGG**	4.03E-06	2.21E-05
*spc11**	AAACAGTGACAGAAACTATTGAGTACGAGG	AGG	4.67E-04	2.90E-02
spc12	CTAGCTTAGATTTTGATACCAATGATCTTA	TTGG	0	7.81E-06
spc13	AATAAAAATAGCACTCCTAATCGTCATCTT	GGCG	0	3.26E-06
spc14	ACTCATTGACGCATACAAAAAGAAAATGAA	A**AGG**	1.21E-05	3.26E-07
*spc14**	CTCATTGACGCATACAAAAAGAAAATGAAA	AGG	1.03E-05	3.26E-07
spc15	ACGTGTAAAGACATATTAGATCGAGTCAAG	G**AGG**	4.48E-07	3.26E-06
*spc15**	CGTGTAAAGACATATTAGATCGAGTCAAGG	AGG	1.92E-04	5.30E-02
spc16	TGTAAAGACATATTAGATCGAGTCAAGGAG	GTTT	0	1.11E-05
spc17	CTAAGAAGTTGTTTCTGTTCAATTTAGAGT	T**AGG**	1.12E-05	0
*spc17**	TAAGAAGTTGTTTCTGTTCAATTTAGAGTT	AGG	1.79E-06	9.77E-07
spc18	AACTGGAATGTTACGAGTGAACTTAAATAT	C**AGG**	1.34E-06	0
spc19	GTATAGGTACAATGTTGACTGATGACTATA	GGCA	0	2.57E-05
spc25	ATCCGTGCCAGTTGGTTGTATTTTTTGATT	G**AGG**	0	0
*spc25**	TCCGTGCCAGTTGGTTGTATTTTTTGATTG	AGG	0	0
spc26	TTATGTTAATTAATCACGGAGCAAAGCGTA	C**AGG**	0	0
*spc26**	TATGTTAATTAATCACGGAGCAAAGCGTAC	AGG	1.02E-04	2.06E-04
spc27	ATCTAGCTTTTTGTACACGAGCATAGCTAC	C**AGG**	0	0
*spc27**	TCTAGCTTTTTGTACACGAGCATAGCTACC	AGG	2.29E-04	1.02E-04
spc36	AAGAAATTATCGAATACTTAAATAAAAAAG	C**AGG**	0	0
*spc36**	AGAAATTATCGAATACTTAAATAAAAAAGC	AGG	0	0

[a] The AGG of the NAGG sequence is bolded
[b] spacer frequency determined by NGS in experiment A
[c] spacer frequency determined by NGS in experiment B

Supplementary Table 2. Importance of different nucleotides to immunity against targets with NAGG PAMs

	***N* of NAGG PAM sequence**				
	**All**	**A**	**C**	**G**	**T**
**mean** [(a)]	1.23	2.14	0.19	0.98	0.96
**variance** [(a)]	2.47	3.19	0.23	1.72	1.76
**n**	679	231	118	133	194
***p* value** [(b)]		2.35E-11	8.39E-39	0.06	0.02
	**last nucleotide of seed sequence**				
	**All**	**A**	**C**	**G**	**T**
**mean** [(a)]	1.23	1.13	1.14	1.63	1.15
**variance** [(a)]	2.47	2.18	1.92	3.66	2.29
**n**	679	249	109	123	199
***p* value** [(b)]		0.39	0.55	0.03	0.52
	**last nucleotide of seed-*N* of NAGG PAM sequence**				
	**All**	**AA**	**CC**	**GG**	**TT**
**mean** [(a)]	1.23	2.17	0.69	2.47	1.71
**variance** [(a)]	2.47	2.82	1.39	4.34	2.67
**n**	679	103	16	22	69
***p* value** [(a)]		3.62E-07	0.10	0.01	0.02

[(a)] Mean and variance of targeting enrichment ratios obtained using NGS data of spacer sequences after phage infection of the NAGG spacer library (Supplementary Fie 1).
[(b)] obtained by performing a Welch's *t*-test, two-sample assuming unequal variances, comparing all sequences ("All") to the specified target sequences.

SUPPLEMENTARY DATA FILES

Supplementary Data File 1. Analysis of spacer sequences obtained using next-generation sequencing.

Supplementary Data File 2. Oligonucleotides, plasmids, strains and phages used in this study.
